# Sounds of Healthy Aging: Assessing Everyday Cognitive Activity From Real-Life Audio Data

**DOI:** 10.1093/geroni/igaa057.2119

**Published:** 2020-12-16

**Authors:** Burcu Demiray, Minxia Luo, Matthew Grilli

## Abstract

The healthy aging model of the World Health Organization (2015) highlights the value of assessing and monitoring everyday activities in understanding health in old age. This symposium includes four studies that used the Electronically Activated Recorder (EAR), a portable recording device that periodically collects sound snippets in everyday life, to assess various real-life cognitive activities in the context of healthy aging. The four studies collected over 100,000 sound snippets (30-seconds long) over a few days from young and older adults in the US and Switzerland. Participants’ speech in the sound snippets were transcribed and coded for different cognitive activity information. Specifically, Haas and Kliegel have investigated the “prospective memory paradox” by examining the commonality and differences in utterances about retrospective and prospective memory failure in young and older adults’ everyday conversations. Demiray and colleagues investigated the relation between autobiographical memory functions and conversation types in young and older adults in relation to well-being. Luo and colleagues have identified the compensatory function of real-world contexts in cognitive aging: Their study showed that older adults benefited from talking with their spouse in producing complex grammatical structures. Finally, Polsinelli and colleagues found robust associations between language markers (e.g., prepositions, more numbers) and executive functions, highlighting the potential use of spontaneous speech in predicting cognitive status in healthy older adults. Finally, Prof. Matthew Grilli will serve as a discussant and provide an integrative discussion of the papers, informed by his extensive work on clinical and cognitive neuroscience of memory in relation to real-life contexts.

